# The effects and acceptability of different exercise modes on glycemic control in type 2 diabetes mellitus: A protocol for systematic review and network meta-analysis

**DOI:** 10.1097/MD.0000000000023963

**Published:** 2021-01-22

**Authors:** Yuanlong Shen, Lina Yu, Zhen Hua, Ningxin Jia, Yanan Zhou, Xiaosheng Dong, Meng Ding

**Affiliations:** a College of Physical Education, Shandong Normal University; b Shandong Provincial Qianfoshan Hospital, The First Hospital Affiliated With Shandong First Medical University; c School of Physical Education, Shandong University, Jinan, Chian.

**Keywords:** exercise, network meta-analysis, type 2 diabetes mellitus

## Abstract

**Introduction:**

Exercise has been believed to have positive effects on blood glucose control in patients with type 2 diabetes mellitus. However, few medical evidences have been found to ascertain which type of exercise has the best effect on blood glucose control in diabetes and which type of exercise is more acceptable. The purpose of this study is to compare the effects and acceptability of different exercise modes on glycemic control in type 2 diabetes patients by using systematic review and network meta-analysis.

**Methods and analysis:**

Relevant randomized controlled trial studies will be searched from PubMed, EMbase, CochraneCENTRAL, CNKI, VIP, and Chinese medical paper libraries. Primary outcome indicators: glycosylated hemoglobin and dropout rate of the research (number of dropouts/numbers of initially enrolled subjects). Secondary outcome measures: fasting blood glucose, body weight, total cholesterol (TC), low-density lipoprotein cholesterol (LDL), high-density lipoprotein cholesterol, triglycerides (TG), diastolic pressure, systolic pressure (SBP). Two reviewers are arranged to screen Title, Abstract, and then review full text to further extract data. Standard meta-analysis and network meta-analysis of the data are performed afterward. Methodological quality assessment is planned to be conducted using Cochrane risk of bias tool. The outcome will be analyzed statistically according to Bayesian analysis methods. After that, subgroup analysis is conducted on the duration of intervention, whether there is supervision of intervention, frequency of intervention per week, age, gender, and medication use.

**Trial registration number:**

PROSPERO CRD42020175181

**Discussion:**

The systematic review and network meta-analysis include evidence of the impact of different exercise modes on blood glucose control in type 2 diabetes mellitus. There are 2 innovative points in this study. One is to conduct a classified study on exercise in as much detail as possible, and the other is to study the acceptability of different exercise modes. The network meta-analysis will reduce the uncertainty of intervention and enable clinicians, sports practitioners, and patients to choose more effective and suitable exercise methods.

**Ethics and dissemination:**

The findings of the study will be disseminated through publications in peer-reviewed journals and scientific conferences and symposia. Further, no ethical approval is required in this study.

## 1 Introduction

As a pandemic, type 2 diabetes has become one of the most serious health concerns and huge burdens over the world. As of 2014, the global adult diabetes morbidity is about 9%.^[1]^ Diabetes is the major cause of cardiovascular disease and its complications are traditionally divided into macrovascular complications and microvascular complications. As the disease progresses, it can cause cardiovascular diseases (including coronary heart disease, peripheral vascular disease, and cerebrovascular disease), kidney disease, retinopathy, diabetic foot, and other complications, and may also increase the risk of musculoskeletal, hepatic, and digestive system diseases, cognitive function and mental health diseases.^[2]^

Exercise is a crucial means to effectively prevent type 2 diabetes in daily life, and it is also a typical preferred treatment strategy. Exercise training is conducive to the control of blood glucose in diabetic patients and thus plays a certain role in enhancing cardiac function.^[2–5]^ Aerobic exercise can reduce glycosylated hemoglobin (HbAlc) index in patients with type 2 diabetes, and also reduce SBP and diastolic pressure, improve pulmonary function, and promote cardiometabolic health.^[6–8]^ Moreover, resistance exercise contributes to regulating blood glucose changes and promoting insulin secretion.^[9]^ Mind-body exercise has a positive influence on improving patients’ blood glucose levels and HbAlc.^[10–12]^Also, high-intensity interval training helps to improve the cardiopulmonary function of patients.^[13]^Aggregate exercise shows a more significant improvement in HbAlc levels.^[14]^

Up to now, based on the traditional meta-analysis of the exercise effect on type 2 diabetes patients, exercise training could effectively improve the early diastolic velocity of the left ventricle and the global systolic longitudinal strain, and significantly improve the maximum oxygen uptake, compared with exercise without intervention.^[4]^ Progressive aerobic exercise is much more able to significantly reduce HbAlclevels than non-progressive aerobic exercise.^[6]^Both muscle hypertrophy training and muscle endurance training improve HbAlc, insulin level and sensitivity, myodynamia, body mass index (BMI), waistline, and fat mass.^[7]^ Compared with moderate-intensity continuous training or no exercise training, aerobic interval training has a better effect on improving the maximum oxygen uptake and reducing the HbAlclevels in clinically stable type 2 diabetes patients.^[8]^ Blood glucose control in patients is related to the intensity of resistance training. High-intensity resistance training can more effectively reduce the HbAlc levels and improve insulin level than that of low-to-medium intensity resistance training.^[9]^ Baduanjinwith conventional treatment can reduce HbAlc, fasting blood glucose (FBG), postprandial blood glucose, TC, TG, and LDL, and raise high-density lipoprotein cholesterol, which is more beneficial to the treatment of patients in contrast Tomer conventional treatment.^[10]^ Furthermore, Tai chi has the effect of reducing HbAlc, FBG, and 2-hour postprandial blood glucose in comparison to inactivity.^[11]^ Yoga exercise has an obvious effect on reducing FBG, postprandial blood glucose, HbAlc, and BMI compared with physical exercise.^[12]^ High-intensity interval training is better than moderate-intensity continuous training or medication in reducing BMI, body fat, HbAlc, fasting insulin, and maximum oxygen uptake.^[13]^

Currently, there are only 2 systematic reviews and network meta-analysis of the effects of different exercise training on blood glucose in type 2 diabetes. One of the studies shows that associated movement has a better effect on the reduction of HbAlc than either supervised aerobic exercise or supervised resistance training. The effect of supervised aerobic exercise on the reduction of FBG, TC, triglyceride, and LDL is significantly better than that of inactivity. Supervised resistance training improves SBP and TC better than inactivity. Supervised aerobic exercise is more effective in improving HbAlc and weight loss than unsupervised aerobic exercise and unsupervised resistance training.^[15]^ Another study found that aerobic exercise is more effective than resistance training in improving HbAlc and fasting blood glucose. Aggregate exercise can significantly reduce the HbAllevel compared with aerobic exercise. By contrast with resistance exercise, associated movement can more effectively reduce the levels of FBG and TC.^[14]^

A network meta-analysis will be exerted to compare the effects and acceptability of different exercise interventions directly or indirectly on blood glucose control in patients with type 2 diabetes. The types of exercise will be divided into specific exercise modes, so that functions and acceptability of different exercise modes could be more accurately and extensively compared and ranked.^[16]^

## 2 Objective

The purpose of this study is to compare the effects and acceptability of different exercise modes on glycemic control in type 2 diabetes patients by using systematic review and network meta-analysis.

## 3 Methods and analysis

### 3.1 Protocol design and registration

Our review has been registered at PROSPERO international prospective register of systematic reviews (ID=CRD42020175181), and the design and reporting of our research programs follow Preferred Reporting Items for Systematic Review and Meta-Analysis Protocols (PRISMA-P)^[17]^ and the Cochrane Collaboration Handbook^[18]^ and is in line with Preferred Reporting Items for Systematic Reviews and Meta-Analyses (PRISMA).^[19]^

### 3.2 Inclusion criteria

If a study meets all the following criteria, it will be included in the network meta-analysis.

#### 3.2.1 Types of studies

The eligible studies shall be a randomized controlled trial, with or without blind method. The minimum duration is 4 weeks for different forms of exercise (eg, aerobic exercise, resistance training, aerobic and resistance training, high-intensity interval training, and mind-body exercise). Animal studies will be excluded. The control group is inactive, and the other 2 groups with different exercise modes will be compared with each other.

#### 3.2.2 Type of participants

Inclusion criteria: patients older than 18 years old with type 2 diabetes will be diagnosed according to the diagnostic criteria of the American diabetes association,^[20]^ regardless of nationality, race, and gender. It also includes patients with type 2 diabetes who have high blood pressure and/or hyperlipidemia, and type 2 diabetes with coronary heart disease.

Exclusion criteria: patients with diabetic complications (diabetic ketoacidosis, infection, diabetic nephropathy, diabetic retinopathy, diabetic foot), chronic kidney disease, type 1 diabetes, and pregnant women and children.

#### 3.2.3 Types of interventions

Studies that meet the above inclusion criteria should include at least one of the following exercise interventions:

1. Aerobic exercise (bicycle, jogging, walking, setting-up exercise, square dance, indoor cycle ergometer, treadmill exercise, brisk walking, swimming, other)^[21,22]^
2. Resistance training (equipment training, dumbbell, resistance band, bodyweight training, combined strength training, full-body resistance training, other)^[23,24]^
3. Aggregate exercise (aerobic exercise + resistance training)^[14]^
4. High-intensity interval training^[25]^
5. Mind-body exercise (tai chi, qigong, yoga, pilates, other)^[26,27]^

**Figure 1:**
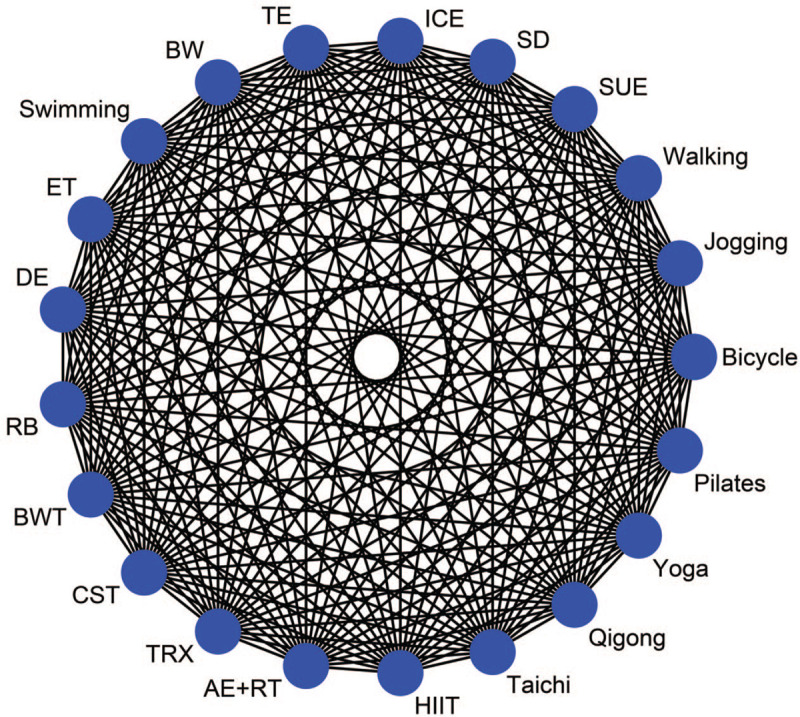
Comparison of all potential pair eligible interventions. AE + RT = Aerobic exercise + Resistance training, BW = Brisk walking, BWT = Body weight training, CST = Combination strength training, DE = dumbbell exercise, ET = equipment training, HIIT = high-intensity interval training, ICE = indoor cycle ergometer, RB = Resistance band, SD = square dance, SUE = setting-up exercise, TE = Treadmillexercise, TRX = Total resistance exercise.

Exercise in the study is combined with other interventions (such as nutrition or psychotherapy), which would be excluded if the effects of the intervention could not be isolated. There must be at least two or more published articles in respect of the required exercise intervention methods. Figure 1 shows a comparison network of eligible interventions.

### 3.3 Outcomes

Primary outcome indicators: HbAlc and dropout rate (number of dropouts/numbers of initially enrolled subjects). Secondary outcome indicators: FBG, body weight, TC, Low-density lipoprotein cholesterol, High-density lipoprotein cholesterol, TG, diastolic pressure, and SBP. These outcomes are also risk factors for cardiovascular disease in patients with type 2 diabetes and are the leading cause of death in patients with type 2 diabetes.

### 3.4 Retrieval strategy

Two reviewers will independently search and check studies, and differences will be discussed and judged by a third reviewer. We will search in PubMed, EMbase, CochraneCENTRAL, CNKI, VIP, and Chinese medical paper library. The published language is in both Chinese, English, and other languages that can be accurately translated into Chinese and English. The retrievals were conducted in both Chinese and English. We will retrieve the original study article in PubMed using the following search term.

1# exercise [full text] or exercise [title/abstract]

2#diabetes [full text] or diabetes [title/abstract] or type 2 diabetes [full text] or type 2 diabetes [title/abstract]

3#aerobic exercise[text] or aerobic exercise [title/abstract] or bicycle [title/abstract] or walking [title/abstract] or jogging [title/abstract] or setting-up exercise [title/abstract] or square dance [title/abstract] or indoor cycle ergometer [title/abstract] ortreadmill exercise [title/abstract] or brisk walking [title/abstract] or swimming [title/abstract]

4#Resistance training [full text] or resistance training [title/abstract] or equipment training [title/abstract] or dumbbell exercise [title/abstract] or resistance band [title/abstract] or bodyweight training [title/abstract] or combined strength training [title/abstract] or total resistance exercise [title/abstract]

5#Aggravate exercise [full text] or aggravate exercise[title/abstract] or aerobic exercise + resistance training [title/abstract]

6#High-intensity interval training [full text] or high-intensity interval training [title/abstract]

7#Mind-body exercise [full text] or mind-body exercise [title/abstract] or Taichi [title/abstract] or Qigong [title/abstract] or Yoga [title/abstract] or Pilates [title/abstract]

8#Glucose[title/abstract] or blood glucose [title/abstract] orfasting blood glucose[title/abstract] orglycosylated hemoglobin[title/abstract] or total cholesterol [title/abstract] or High-density lipoprotein cholesterol [title/abstract] or Low-density lipoprotein cholesterol [title/abstract] or triglycerides [title/abstract] or diastolic pressure[title/abstract] or systolic pressure [title/abstract]

9# randomized controlled trial [full text] or randomized controlled trial [title/abstract]

10# (1# and 2# and 3# and 4# and 5# and 6# and 7# and 8# and 9#)

Besides, we will review the reference list of articles and retrieve studies that may meet the inclusion criteria. It is also reasonable to search for ongoing trials on trial registry sites (such as NIH resources and the WHO international clinical trial registry platform). In addition, it is not limited by year of publication, country, language, the status of publication, or date of publication.

### 3.5 Steps for research retrieval and inclusion

**Figure 2:**
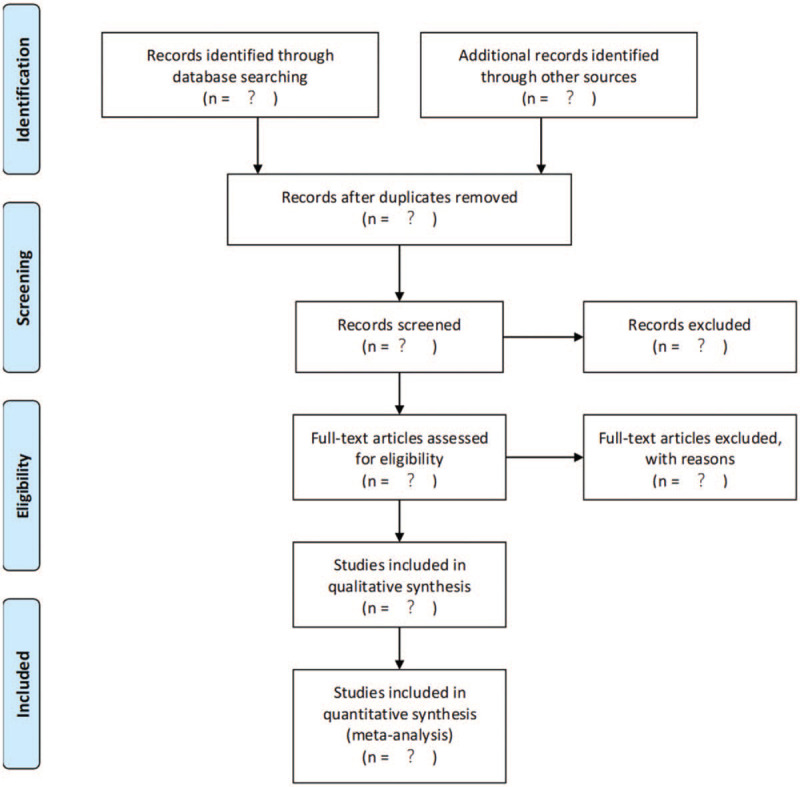
Flow chart of steps for research retrieval and inclusion.

ENDNOTE X9^[28]^ document management software will be applied. First, the retrieved articles are imported into the software for preliminary screening to exclude duplicate documents. Second, 2 reviewers screen the abstracts and titles of the retrieved literature according to the inclusion criteria and exclude the literature that does not meet the criteria. Finally, the full text of qualified research will be obtained, and the reviewers will review the full text again and exclude the literature that does not meet the criteria. The 2 reviewers independently screen and decide, and the differences will be resolved through discussion and consultation. If no agreement can be reached, the third reviewer shall intervene to make a final decision. The flow chart (Fig. 2) outlines the inclusion steps and exclusion reasons for full-text retrieval.

### 3.6 Data extraction

Two reviewers shall independently extract the data and input them into the Excel data extraction table any differences shall be settled by the third reviewer through negotiation. The reviewers will extract the following information from the original study:

1. Research information (name of the first author, journal name, year of publication, random method, and blind method)
2. Sample characteristics (sample size, age, sex, height, weight, BMI, diagnostic criteria for type 2 diabetes, population type)
3. Intervention characteristics (type, frequency, and time of exercise)
4. Outcome measures (glycosylated hemoglobin, research dropout rate, total cholesterol, High-density lipoprotein cholesterol, Low-density lipoprotein cholesterol, triglycerides, diastolic pressure, SBP.)

### 3.7 Risk of bias assessment

It is expected to assess the methodological quality of randomized controlled trials by two reviewers using ROB, The Cochrane Collaboration's tool for assessing risk of bias.^[29]^The sources of bias are discussed from the following seven dimensions:

(1) random sequence generation;
(2) allocation concealment;
(3) concealment of research objects and personnel;
(4) concealment of results measurement;
(5) incomplete result data;
(6) selective reporting results;
(7) other bias.

The risk of bias is judged by 3 levels: low risk, unclear, and high risk. Disputes arising from the evaluation process shall be discussed and negotiated collectively. If no settlement can be reached, a third reviewer shall intervene for settlement.

### 3.8 Processing of missing data

We are going to contact the original author via email or phone to get the missing data, identify the cause, and discuss how to deal with the missing data.

### 3.9 Research rating

GRADE software is used to assess the quality of evidence and make recommendations, and GRADE guidelines are applied to assess the quality of evidence and the strength of key outcome recommendations.^[30]^There are 5 factors affecting evidence rating, namely, research limitations, indirection, inconsistencies, inaccuracy, and publication bias. Three factors increase the level of evidence: high effect, the dose-response relationship, and residual confounding. The quality of initial evidence registration is classified into 4 levels: high, medium, low, and very low. The initial evidence level of the randomized controlled trial is high quality, which will be relegated according to the following predefined criteria: study limitations (the assessment of bias risk is to assign different weights to the low risk, unclear and high risk, and to assess whether to downgrade), indirectness (indirect comparison to show whether the hypothesis is true), inconsistency (inconsistency with any nodes, *I*^2^ > 50% and *P* < .10), inaccuracy (whether 95% CI contains significant benefits or harms) and publication bias (significant evidence of small study effects).

### 3.10 Data statistics

#### 3.10.1 General data description

It is necessary to design descriptive statistical data tables to describe study characteristics and variables (such as years of study, the average age difference of subjects, gender, intervention measures, etc.). A network evidence plot is used to present a direct comparison between groups with different exercise interventions and the control group. The size of the nodes is equal to the number of intervention groups, and the thickness of the edges is equal to the number of studies directly compared. We decide to use the network contribution plot to calculate the percentage of each direct comparison to the results of the corresponding comparison network meta-analysis and the percentage of each direct comparison to the entire network.

#### 3.10.2 Standard meta-analysis and network meta-analysis

For each outcome measurement, we will perform a paired meta-analysis. The standard meta-analysis will be used to directly compare all interventions with at least 2 intervention studies. For dichotomous outcome indicators, the effect value OR is used for evaluation, and for continuous outcome indicators, the MD and SMD are used for evaluation. *I*^2^ and *P* values are employed to assess the heterogeneity of the study.^[31]^*P* > .1 and *I*^2^ > 50% indicate heterogeneity, which entails identification of the source of heterogeneity and reduction of heterogeneity through sub-group analysis. Random effect model will be employed for statistics given that the source of heterogeneity cannot be obtained or statistical heterogeneity exists.^[32,33]^The forest graph shows the effect value and 95%CI. If there is considerable heterogeneity, we will make a descriptive analysis.^[34]^

Network meta-analysis is an extension of standard meta-analysis, which integrates direct comparative evidence and indirect or mixed comparative evidence. The Markov Chain Monte Carlo method based on Bayesian framework is used for calculation and statistics,^[35]^ and R language software is used for implementation.^[36]^ The measures of effect are demonstrated using the effect value OR or the MD and 95% confidence interval. We will also use the value of the surface under the cumulative ranking curve to sort each exercise mode. The greater the value of surface under the cumulative ranking curve is, the more effective the intervention shall be.^[37]^

#### 3.10.3 Estimation of similarity

There is a certain similarity between the 2 indirectly compared test sets, and the similarity of clinical features and methodological features is mainly evaluated, which is mainly dependent on opinions of epidemiologists or clinical experts, as well as baseline data such as patients and trial design. Currently, there is no statistical test method for similarity.

#### 3.10.4 Inconsistency assessment and treatment

Inconsistencies are defined as inconsistencies between direct and indirect evidence, including loop-based inconsistencies (direct and indirect comparisons within the loop) and design inconsistencies (indirect comparisons between 2 arms and multiple arms). If there are only 2-arm tests in the study, only loop-based inconsistencies are evaluated. We will split nodes to evaluate local inconsistencies in the network meta-analysis model. If *P* > .05, there is consistency; if *P* < .05, there is inconsistency.^[38]^

#### 3.10.5 Subgroup analysis and sensitivity analysis

Subgroup analysis is performed on the factors that may lead to heterogeneity (The severity, course, and BMI of diabetes). Sensitivity analysis will be performed by excluding the studies included in the analysis item by item and comparing the results.

#### 3.10.6 Publication bias

When the number of studies is large enough (at least 10 studies), it is possible to use the corrected comparison funnel plot to evaluate publication bias, with the horizontal axis indicating the difference between the effect value in an effect comparison and the combined effect value of all similar comparisons, and the vertical axis indicating the standard error of the effect value.^[39]^If there is no small sample effect, the funnel plot will be symmetric around the zero lines.

### 3.11 Patient and public involvement

No patients or public involvement will be involved in this study.

## 4 Discussion

Exercise is an effective treatment for patients with type 2 diabetes. Exercise can not only regulate blood glucose levels, reduce HbAlc, improve dyslipidemia, but also reduce weight, regulate mood, and improve cardiopulmonary function and quality of life.

The innovation of our research lies in:

(1) a more comprehensive and detailed classification of exercise modes, and
(2) the acceptability of different exercise modes.

Besides, we will rank the exercise modes in the study, to provide more intuitive judgment for clinicians, hoping that it can provide clinical suggestions to practically help patients with type 2 diabetes choose the most effective exercise modes.

There are limits in this study, mainly for the heterogeneity, inconsistency, and publication bias of the study. To reduce the heterogeneity of the method, we will choose the random effect model and summarize the effect indicator, which can eliminate some heterogeneity. If the number of studies is small and heterogeneity is not good, we can correct heterogeneity through subgroup analysis and Meta regression. As to inconsistencies, we will explore the causes of inconsistencies and use network meta-regression or network subgroup analysis to minimize inconsistencies. For the original research with large publication bias, it is possibly resolved by contacting the original author to provide relevant data as far as possible. If the bias in the sensitivity analysis seriously affects the results, it will be marked and then truthfully reported.

## Author contributions

**Conceptualization:** Meng Ding.

**Data curation:** Yuanlong Shen, Lina Yu, Ningxin Jia, Yanan Zhou.

**Methodology:** Xiaosheng Dong, Meng Ding.

**Writing – original draft:** Yuanlong Shen.

**Writing – review & editing:** Yuanlong Shen, Lina Yu, Zhen Hua, Meng Ding.
